# Miller-Fisher Syndrome Presenting as Facial Diplegia With COVID-19 Co-Infection

**DOI:** 10.7759/cureus.17060

**Published:** 2021-08-10

**Authors:** Cuong Tran, Blake Drury, Ho-Wang Yuen, Johanna Rosenthal, Michael M Neeki

**Affiliations:** 1 Emergency Medicine, Arrowhead Regional Medical Center, Colton, USA; 2 Internal Medicine, Arrowhead Regional Medical Center, Colton, USA

**Keywords:** covid-19, miller-fisher syndrome, facial diplegia, facial palsy, guillain-barré syndrome

## Abstract

Coronavirus disease 2019 (COVID-19) has reportedly been associated with various neurological manifestations, including unilateral facial palsy and, very rarely, facial diplegia. We present a unique case of Miller-Fisher Syndrome (MFS), a variant of Guillain-Barré Syndrome (GBS) that was noted in conjunction with a COVID-19 infection. In this case, a patient presented with bilateral facial palsy, dysarthria, right-sided hemiparesis, ataxia, and the confirmation of SARS-CoV-2 infection. His computed tomography (CT) scan of the brain and serology test results did not support alternate etiologies for facial palsy. His cerebrospinal fluid (CSF) studies demonstrated albuminocytologic dissociation, which was consistent with the diagnosis of MFS and further supported by his ataxia and ophthalmoplegia. A five-day course of intravenous immunoglobulin (IVIG) therapy combined with physical, occupational, and speech therapy improved his recovery.

## Introduction

Severe acute respiratory syndrome coronavirus 2 (SARS-CoV-2), which causes coronavirus disease 2019 (COVID-19), is primarily identified by a respiratory syndrome [1]. However, as the number of cases continues to rise during the current global pandemic, there is an increase in association between COVID-19 and various abnormal neurological manifestations and complications. These symptoms range from anosmia, ageusia, and headache to more severe presentations of stroke, seizures, and encephalopathy [2-3].

The exact mechanisms and pathophysiology for COVID-19-induced neurological symptoms remain largely unknown and remain to be elucidated. At the cellular level, it is suspected that the SARS-COV-2 virus binds to the human angiotensin-converting enzyme 2 (hACE2) [4]. These receptors are abundant in the lung epithelia but are also present in the small intestines and in the brain at the vascular and neuronal levels [5-6]. It has been suggested that compared to the prior severe acute respiratory syndrome (SARS), which was responsible for the outbreak in 2003, the novel SARS-COV-2 binds to cell membranes expressing hACE2 with greater affinity, resulting in its increased neuro-invasive potential and neurological sequelae [1,7].

The involvement of peripheral nerves, neuromuscular junctions, and the musculoskeletal system have been reported and resulted in the presentations of disease processes such as myositis, rhabdomyolysis, and Guillain-Barré Syndrome (GBS), among others [8]. Here, we present a patient with the SARS-COV-2 virus infection who presented with a unique finding of facial diplegia and subsequently was diagnosed with a variant of GBS. This study was approved by the Institutional Review Board at Arrowhead Regional Medical Center with approval number 21-13.

## Case presentation

A 42-year-old male presented to the emergency department (ED) with acute onset of right lower extremity weakness, decreased right-sided facial sensation, slurred speech, and headache. His symptoms began 12 hours prior to arrival. His vital signs were notable for blood pressure (BP) of 153/113 mmHg, heart rate (HR) of 116 beats per minute, respiration rate (RR) of 28 per minute, temporal temperature (temp) of 97.5° (F), and basal oxygen saturation of 97% (SaO2) on room air. His past medical history was significant for non-insulin-dependent diabetes mellitus. His physical exam was notable for muscular strength rated 4/5 in the right lower extremity but 5/5 in all other extremities. He had a decrease in right-sided facial sensation and slight dysarthria. The remainder of his neurological exam was otherwise unremarkable.

**Figure 1:**
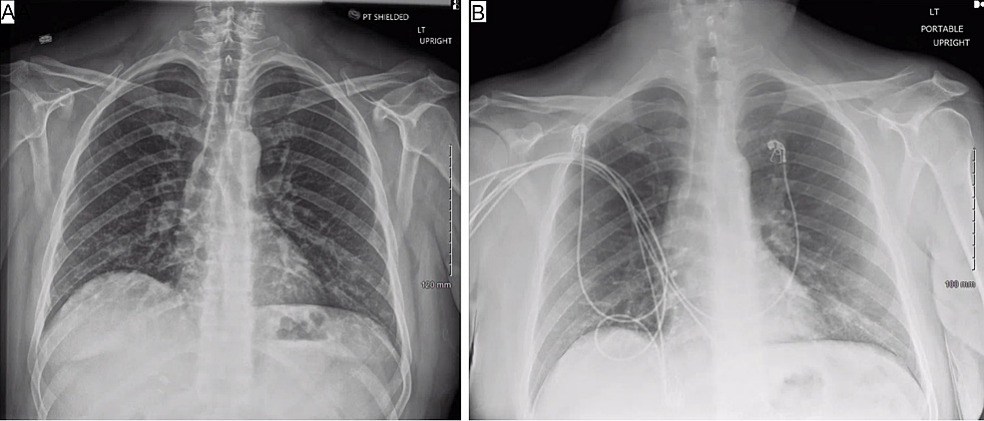
Chest X-ray of the patients at the initial visit (Figure 1A) and second visit (Figure 1B)

Since his acute onset of symptoms was concerning for cerebrovascular events, he immediately underwent computed tomography (CT) scan of his brain without contrast followed by a CT scan with intravenous contrast, which did not reveal any emergent intracranial or intravascular abnormality (Figure 1, panel A). Non-contrast magnetic resonance imaging (MRI) of the brain was pursued as part of the investigation and did not show any abnormalities. The patient was also screened for COVID-19 by a nasopharyngeal swab using reverse transcriptase-polymerase chain reaction assay (BioFire Diagnostics, Salt Lake City, UT), which was positive. As part of the stroke workup, the patient had a chest X-ray (CXR) done, which showed bibasilar infiltrates; however, he did not present with any respiratory symptoms. While under close monitoring in the ED, his focal neurological weakness of the right lower extremity improved. After a period of observation along with evaluation by the neurology team, he was discharged home with a planned follow-up with his primary physician.

Four days following the initial visit, he was transported back to the same ED by emergency medical services (EMS) with more pronounced dysarthria, facial weakness, right lower extremity weakness, ataxia, and diplopia. Repeat vitals was notable for a BP of 140/110 mmHg, HR of 101 BPM, RR of 20 per min, and oxygen saturation in the arterial blood (SaO2) of 98% on room air. He had bilateral ptosis and was unable to raise his eyebrows on either side or smile. His speech was comprehensible but notable for mild dysarthria. He had noticeable difficulty with his left lateral gaze. His strength was 4/5 in his right upper and lower extremities compared to full strength on the left side, and he was able to stand with minimal assistance. Both his dysdiadochokinesia and Romberg tests were normal. His reflexes were 1+ throughout, and the remainder of his neurological exam was unremarkable.

A repeat CXR revealed persistent bibasilar infiltrates compared to his prior visit (Figure 1, panel B). The repeat CT of his brain without and with IV contrast did not demonstrate any new acute intracranial or intravascular abnormalities. His laboratory analysis was notable for white blood cell (WBC) count of 10.3 th/uL, hemoglobin (Hb) level of 16.0 g/dL, hematocrit (HCT) of 43%, and platelet count of 612 th/uL (120-360). There were mild electrolyte derangements, including sodium of 133 meq/L (135-148), potassium of 3.0 meq/L (3.5-5.5), chloride of 97 meq/L (98-110), phosphorus of 1.8 mg/dL (3.4-4.4), carbon dioxide of 21 mmoles/L (24-34), and serum glucose of 222 mg/dL. Additional labs included mild transaminitis with aspartate transaminase (AST) of 57 u/L (5-40), alanine transaminase (ALT) of 112 u/L (5-40), and elevated total bilirubin of 1.5 mg/dL (0.0-1.2). In addition, procalcitonin elevation was noted at 0.17 ng/mL (<0.08) but his C-reactive protein level was within normal limits.

He was treated with dexamethasone 10 mg intravenous (IV) in the ED as well as electrolyte replacement for hypokalemia. After further discussion with the neurologist and the patient himself regarding his focal neurological deficits, a lumbar puncture was performed. Cerebrospinal fluid (CSF) studies noted an albumin of 200 mg/dL (normal <50 mg/dL), which suggested increased permeability consistent with GBS, and an elevated CSF protein level of 101 mg/dL (15-45 mg/dL) with a CSF WBC count of 3/uL, indicating albuminocytologic dissociation. In addition, the CSF studies were notable for an elevated glucose level at 105 (40-70 mg/dL) but normal cytology analysis and cultures. Subsequently, he was admitted to the hospital and remained in the ward for a period of eight days for further evaluation and close observation.

**Figure 2:**
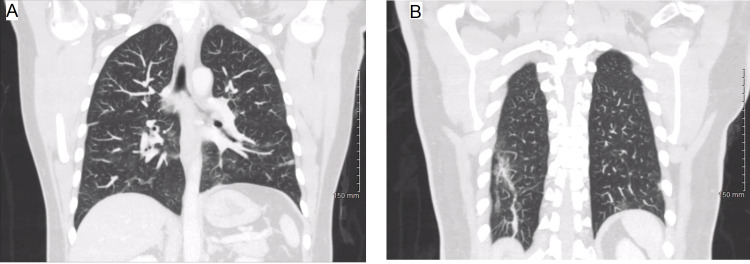
CT scan during the second visit indicates scattered areas of ground-glass opacity and bilateral lower lobe linear atelectasis

Once more, he underwent brain MRI, which failed to demonstrate any abnormal pathology. While inpatient, a CT scan of the patient's thorax with IV contrast demonstrated scattered areas of ground-glass opacities suggestive of hypersensitivity pneumonitis versus atypical pneumonia, without evidence of a mediastinal mass to suggest thymoma (Figure 2 panels A-B). Serologies and extensive CSF studies did not support Lyme disease, West Nile virus, HIV, or syphilis, and did not reveal elevated myelin protein or oligoclonal bands. He was given a pyridostigmine trial consisting of 60 mg per mouth (PO) every six hours for 24 hours for possible myasthenia gravis without noticeable improvement in symptoms.

Based on his constellation of symptoms, including ataxia, ophthalmoplegia, and evidence of albuminocytologic dissociation on CSF studies, he was diagnosed with Miller Fischer Syndrome (MFS). The patient subsequently underwent intravenous immunoglobulin (IVIG) at 0.45 g/kg/day. He received a five-day course of IVIG, and at the end of the first day of IVIG infusion, he demonstrated the ability to raise his eyebrows for the first time since admission. He was further supported with physical, occupational, and speech therapy and showed significant improvement in his lower extremity strength, facial weakness, and speech; he was able to ambulate independently at the time of discharge. One month following discharge from the hospital, the patient’s facial weakness fully recovered, and his diplopia had resolved. His strength had improved nearing baseline, and he was able to ambulate independently but sometimes experienced ‘shakiness’ in his lower extremities during prolonged activities. He continued to experience very mild residual numbness on both sides of his face, specifically his cheeks and the area below his lips but clarifies that the paresthesia had significantly improved compared to his initial presentation to ED.

## Discussion

In general, facial diplegia is exceptionally rare, representing only 0.3% to 2.0% of all cases of facial nerve palsy [9]. It is suggested to be caused by many conditions, including sarcoidosis, Lyme disease, GBS, Epstein-Barr virus, brain tumors, and leukemia [9]. While the literature is scant regarding facial diplegia as a neurological complication of SARS-CoV-2, Caamaño and colleagues suggested it could occur in an atypical variant of GBS [10]. Overall, GBS accounts for 3% of reported bilateral facial palsy cases and is typically preceded by a viral upper respiratory or gastrointestinal infection [11]. MFS is a clinical variant of GBS, typically characterized by ophthalmoplegia, ataxia, and areflexia. In addition, it may include symptoms such as dysarthria, dizziness, and cranial nerve involvement, which can lead to facial paresis. Although it is one of the rarer neurological sequelae, there have been an increasing number of case reports linking GBS, an immune-mediated polyneuropathy, with COVID-19 infection and even fewer reported cases associated with the MFS variant [12].

At the time of writing, several cases have described GBS to be associated with a preceding or concurrent COVID-19 infection, but only two cases presented with facial diplegia [13-14] and only eight prior cases reports were associated with MFS [15-22] These details of prior reported cases, including presenting neurological and respiratory symptoms, diagnosis, treatment, and outcome, are documented in Table 1.

**Table 1:** Cases of MFS associated with recent or concurrent COVID-19 infection *Progressive improvement indicates deficits partially improved prior to discharge;**Patient offered admission for IVIG treatment if the condition worsened MFS=Miller Fischer Syndrome; LE=lower extremity; UE=upper extremity; LUE=left upper extremity; RLE=right lower extremity; MRI=magnetic resonance imaging; CT=computed tomography; IVIG=intravenous immunoglobulin; INO=internuclear ophthalmoplegia; b/l=bilateral; CN=cranial nerve

Author	Presenting symptoms	Additional exam findings and lab findings	Anti-GQ1B	Abnormalities on CT or MRI	Treatment	Outcome
Lantos [15]	Left ptosis, blurry vision, bilateral LE paresthesia	Areflexia	Negative	MRI: enlargement, T2 hyperintensity, and enhancement of CN III	IVIG x5d (days)	Progressive improvement*
Gutiérrez-Ortiz [16]	Anosmia, ageusia, right INO, right fascicular CN III palsy, ataxia	Areflexia; albuminocytological dissociation	Positive	N/A	IVIG x5d	Complete recovery (except anosmia and ageusia)
Reyes-Bueno [17]	Paresis of left external rectus muscle with horizontal diplopia when looking left; b/l facial paresis, b/l LE weakness	Areflexia; albuminocytological dissociation	Negative	N/A	IVIG x5d	Progressive improvement
Ray [18]	Diplopia, ataxia, right perioral paresthesia distal b/l UE paresthesia	Areflexia; albuminocytological dissociation	N/A	N/A	None**	Progressive improvement
Senel [19]	Diplopia, ataxia, paresthesia distal b/l UE	Areflexia; albuminocytological dissociation	Negative	N/A	IVIG x5d	Complete recovery
Manganotti [20]	Ageusia, diplopia, facial paresthesia, ataxia, LUE dysmetria, mild lower facial weakness, mild left facial paresthesia	Areflexia	Negative	N/A	IVIG x5d	Complete recovery
Fernández-Domínguez [21]	Ataxia, blurred vision	Albuminocytological dissociation	Negative	MRI: None	IVIG x5d	Progressive improvement
Dinkin [22]	Left ptosis, diplopia, b/l distal leg paresthesia, ataxia; subjective fever, cough, myalgias	Hyporeflexia	Negative	MRI: enhancement T2 hyperintensity, enlargement of L CN III	IVIG x3d	Progressive improvement
Present study	RLE weakness, facial diplegia, facial paresthesia, dysarthria, ataxia, diplopia; headache	Albuminocytological dissociation	N/A	CT/MRI: None	IVIG x5d	Complete recovery

Traditionally, MFS is thought to be caused by an aberrant autoimmune response through molecular mimicry of a peripheral nerve antigen and viral polysaccharides components, causing an inflammatory process [23]. This is a rare event often found in association with anti-GQ1b ganglioside antibodies, but its absence does not exclude MFS [24]. Numerous studies mentioned in Table 1 had undetectable levels of serum anti-ganglioside antibodies, including anti-GQ1b. Beyond the theory of hACE2 involvement, it is unclear exactly how the disease process of MFS is induced in individuals who have COVID-19. Treatment for MFS in this context is not largely different from the standard of care. Given the risk of respiratory involvement with MFS, where invasive airway interventions, such as intubation, may be required, patients are typically admitted for close observation and treated with either IVIG or plasmapheresis, and the prognosis is reported to be typically good [23].

This patient presented with bilateral facial palsy, dysarthria, diplopia, right-sided weakness, and evidence of SARS-CoV-2 infection without the typical respiratory sequelae. The presentation of bilateral facial palsy in the setting of COVID-19 infection was clinically unique. Although cases of GBS associated with COVID-19 are becoming increasingly common, MFS with facial diplegia, as seen in our patient, is still quite rare. In this case, IVIG was found to be effective in treating MFS in a COVID-19 positive patient with complete neurological recovery.

There are a few limitations during the management course for the patient. First, the diagnosis of MFS is typically based on detailed history and clinical exam with the additional support of the laboratory tests. While the relevant antibody tests (such as anti-ganglioside) can be conducted in few institutions, at our institution, the results would have taken approximately five to seven days. As a result, the hospitalist team decided to forego further testing when deciding on time-sensitive inpatient treatment. Second, the nerve conduction study (NCS)/electromyography (EMG) studies were recommended by the neurology team and considered by the inpatient team, however, they were ultimately not pursued, in part, because there was enough clinical evidence to support the diagnosis, the limited resources during the COVID-19 pandemic, and the patient’s rapid improvement with IVIG. Last, the Brighton Criteria can serve as an additional tool to assist in diagnosing GBS and help distinguish between low- and high-risk patients. The Brighton Criteria is based on the diagnostic criteria, including extremity weakness, CSF values, NSC findings, the course of the disease, and the absence of an alternative diagnosis [25]. As a result, it cannot be calculated without results from NCS/EMG for our particular MFS case.

## Conclusions

As cases of COVID-19 are increasing, clinicians should consider the diagnosis of COVID-19 in patients who present with neurological complaints with or without respiratory symptoms. This patient presented with rare neurological symptoms that required an effective team of multidisciplinary specialists for rapid identification and the prompt initiation of effective treatment.
